# An interspecific barberry hybrid enables genetic dissection of non-host resistance to the stem rust pathogen *Puccinia graminis*

**DOI:** 10.1093/jxb/ery066

**Published:** 2018-02-26

**Authors:** Radhika Bartaula, Arthur T O Melo, Bryan A Connolly, Yue Jin, Iago Hale

**Affiliations:** 1 Department of Agriculture, Nutrition, and Food Systems, University of New Hampshire, Durham, NH, USA; 2 Department of Biology, Framingham State University, Framingham, MA, USA; 3 USDA-ARS Cereal Disease Laboratory, St. Paul, MN, USA

**Keywords:** Barberry, genotyping by sequencing, non-host resistance, stem rust, wheat

## Abstract

Stem rust, caused by *Puccinia graminis* (*Pg*), remains a devastating disease of wheat, and the emergence of new *Pg* races virulent on deployed resistance genes fuels the ongoing search for sources of durable resistance. Despite its intrinsic durability, non-host resistance (NHR) is largely unexplored as a protection strategy against *Pg*, partly due to the inherent challenge of developing a genetically tractable system within which NHR segregates. Here, we demonstrate that *Pg’s* far less studied ancestral host, barberry (*Berberis* spp.), provides such a unique pathosystem. Characterization of a natural population of *B.* ×*ottawensis*, an interspecific hybrid of *Pg*-susceptible *B. vulgaris* and *Pg*-resistant *B. thunbergii* (*Bt*), reveals that this uncommon nothospecies can be used to dissect the genetic mechanism(s) of *Pg*-NHR exhibited by *Bt*. Artificial inoculation of a natural population of *B.* ×*ottawensis* accessions, verified via genotyping by sequencing to be first-generation hybrids, revealed 51% susceptible, 33% resistant, and 16% intermediate phenotypes. Characterization of a *B.* ×*ottawensis* full sib family excluded the possibility of maternal inheritance of the resistance. By demonstrating segregation of *Pg*-NHR in a hybrid population, this study challenges the assumed irrelevance of *Bt* to *Pg* epidemiology and lays a novel foundation for the genetic dissection of NHR to one of agriculture’s most studied pathogens.

## Teaser

This study demonstrates for the first time the feasibility of dissecting the genetic mechanism of non-host resistance to the stem rust pathogen in its ancestral host genus *Berberis*.

## Introduction

Stem rust, caused by the fungal pathogen *Puccinia graminis* (*Pg*), is one of the most devastating diseases of wheat and other small grains, and is responsible for severe epidemics and major recurring yield losses worldwide (Leonard and Szabo, 2005). The threat of *Pg* to global food security is further enhanced by its ability rapidly to evolve new forms and combinations of virulence (Pretorius *et al.*, 2000). Since the pioneering work of Dr Elvin Stakman nearly 90 years ago (see Christensen, 1984), tremendous effort has been made by a global community of researchers to identify and deploy genetic sources of *Pg* resistance in wheat cultivars. Despite these efforts, the protection conferred by the vast majority of resistance genes has been temporary, or non-durable, due to evolving virulence (Singh *et al.*, 2015). With concern rekindled over the emergence and spread of new virulent stem rust races, most notably the Ug99 family of races radiating out of East Africa, the search for novel sources of durable resistance has come to be considered essential to achieving long-term wheat security (Stokstad, 2007; Ayliffe *et al.*, 2008).

Non-host resistance (NHR), in which an entire plant species is resistant to all genetic variants of a pathogen species, is the most common type of resistance exhibited by plants (Lipka *et al.*, 2010). Given its intrinsic durability and efficacy across a broad range of pathogens (Thordal-Christensen, 2003; Mysore and Ryu, 2004), NHR presents a compelling strategy for achieving long-term rust control in wheat. In his dream for tomorrow, Dr Norman Borlaug envisaged the eventual transfer of *Pg*-NHR from rice to wheat, forever solving via biotechnology the historic rust problem plaguing one of humanity’s most important staple crops (Borlaug, 2000). When simply inherited, the proof of concept for such a visionary transfer of NHR between species has been demonstrated [e.g. maize gene *Rxo1* for bacterial streak disease of rice (Zhao *et al.*, 2005)], but some studies suggest that *Pg*-NHR may not be simply inherited (Cheng *et al.*, 2012).

Considerable effort has been made to understand the response to rust pathogens using various non-host and intermediate host pathosystems, including *Uromyces vignae*, *Puccinia triticina*, and *P. striiformis* on the model plant *Arabidopsis thaliana* (Mellersh and Heath, 2003; Shafiei *et al.*, 2007; Cheng *et al.*, 2013); *P. graminis*, *P. triticina*, *P. striiformis*, *P. hordei*, *P. sorghi*, and *Melampsora lini* on rice (Ayliffe *et al.*, 2011); *P. striiformis* on broadbean (Cheng *et al.*, 2012); *P. hordei* and *U. fabae* on wheat (Prats *et al.*, 2007; Zhang *et al.*, 2011); and *P. striiformis*, *P. triticina*, *P. hordei*, *P. coronata*, *P. recondite*, *P. hordei-secalini*, and *P. persistens* on barley (Atienza *et al.*, 2004; Jafary *et al.*, 2008; Dawson *et al.*, 2016). While the specific mechanisms underlying NHR to rust fungi remain unknown, progress has been made in understanding the basal host defense responses of NHR under pathogen pressure. The current body of knowledge, based largely on histological and cytological studies, suggests that NHR involves multiple mechanisms, including callose deposition, production of reactive oxygen species, phytoalexin synthesis, salicylic acid signaling, and jasmonic acid signaling (Perumalla and Health, 1989; Ayliffe *et al.*, 2011; Zhao *et al.*, 2016). Despite a growing understanding of such basal mechanisms of some forms of NHR, however, little is known about the genetics underlying such responses. Since all of the individuals in a non-host plant species are, by definition, resistant to the pathogen, relevant genetic analyses are difficult to perform. Simply stated, in order to study the genetics of this type of resistance, a genetically tractable system segregating for NHR is required.

Within the highly diverse *Berberis* genus, numerous species are known to function as competent alternate (or sexual) hosts to *Pg* (Jin, 2011; Zhao *et al.*, 2013), but others do not. For example, European barberry (*B. vulgaris* L.) is susceptible to *Pg* infection, but Japanese barberry (*B. thunbergii* DC.) is identified as a non-host, with no infection observed either under natural conditions or through extensive laboratory testing (Levine and Cotter, 1932). The association of *B. vulgaris* with *Pg* has been implicated in the wheat stem rust pathosystem for centuries, as evidenced by the existence of *B. vulgaris* eradication laws as far back as the 1600s (Zadoks and Bouwman, 1985). From 1918 to 1974, a massive *B. vulgaris* eradication program was undertaken by the US government as a means of controlling wheat stem rust (Peterson, 2013). Under that program, the largest plant eradication effort in history, >500 million common barberry plants were destroyed throughout the North Central Plains of the USA (Peterson, 2003). In contrast, cultivars of *B. thunbergii* continue to this day to be sold as part of a multi-million dollar ornamental landscape industry (Lubell *et al.*, 2008), provided their resistance to *Pg* is confirmed by the USDA Cereal Disease Laboratory through its long-running barberry testing program (Silander and Klepeis, 1999). In the northeastern USA, outside the boundary of the 20th century federal barberry eradication zone, both *B. vulgaris* and *B. thunbergii* are found in great abundance, to the extent that both are considered invasive species (Silander and Klepeis, 1999; Mehrhoff *et al*., 2003). When the two species co-occur, they can hybridize to produce the relatively rare nothospecies *B.* ×*ottawensis*, and several natural populations of this interspecific hybrid have been documented in the region in recent years (Connolly *et al.*, 2013; Hale *et al.*, 2015; Van Splinter *et al.*, 2016).

Despite the evolutionary relationship between some barberry species and *Pg*, and despite wheat stem rust being one of the most intensively researched plant diseases, no attempt has been made to understand the genetic mechanism of *Pg*-NHR exhibited by some *Berberis* spp. It is hypothesized that the modern-day macrocyclic, heteroecious species of *Pg* evolved from a progenitor that existed in aecial form, parasitizing dicot ancestors of the Berberidaceae prior to its host expansion to the grasses (Leppik, 1961; Wahl *et al.*, 1984). It is thus of interest to investigate the mechanism of NHR exhibited by descendants of the ancestral hosts of cereal rust pathogens. Since *B. vulgaris* is a competent host of *Pg* and *B. thunbergii* is not, their interspecific hybrid presents a unique system for studying the genetic mechanism(s) of the apparent *Pg*-NHR in *B. thunbergii*. For this study, we utilized a natural population of *B.* ×*ottawensis* to determine if indeed the nothospecies can be used toward this end, thereby providing insight into mechanisms of NHR that may inspire novel strategies of stem rust resistance in wheat.

## Materials and methods

### Study taxa and field survey

**Fig. 1.**
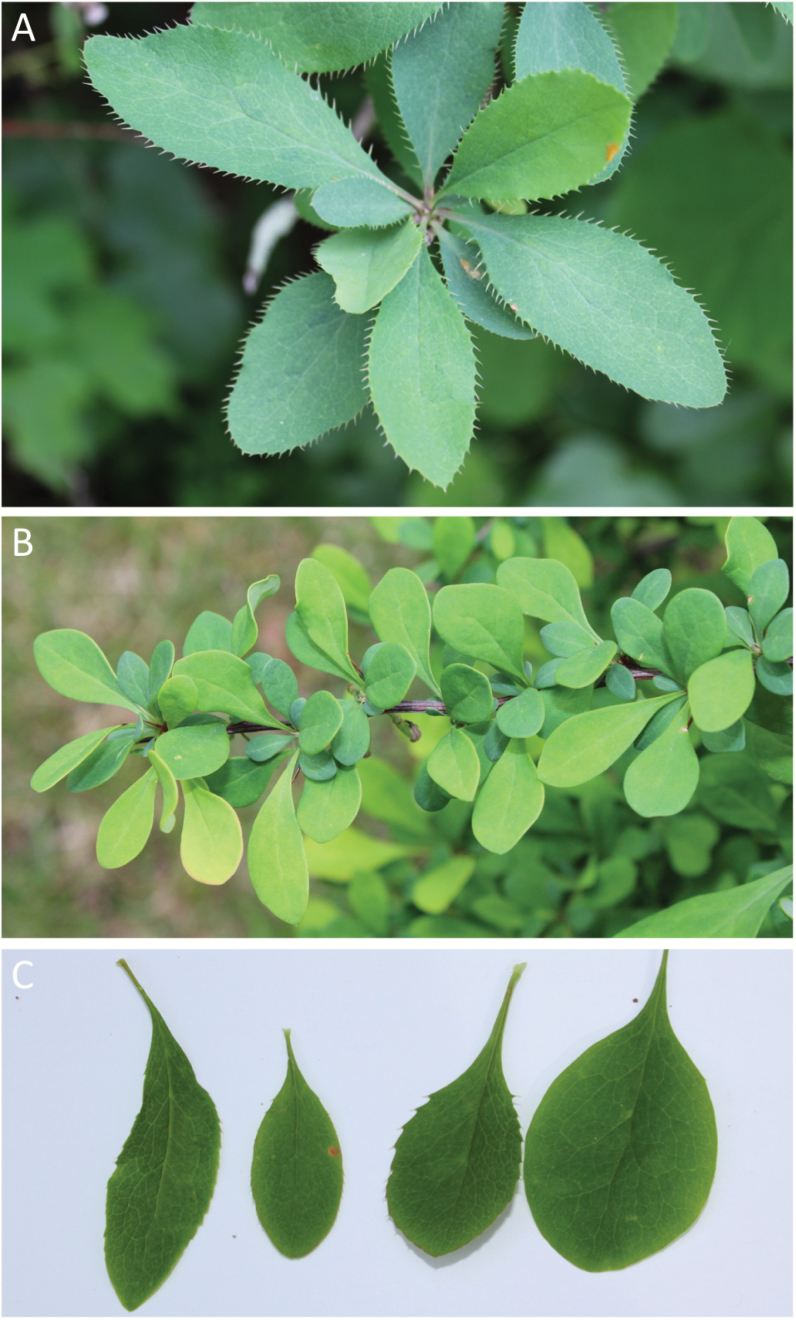
Leaf morphologies of the three *Berberis* taxa in the study. (A) 2–5 cm long obovate to obovate–oblong leaves with highly serrated margins (>50 serrations) typical of *B*. *vulgaris*; (B) 1–4 cm long, entire leaves typical of *B*. *thunbergii*; and (C) variations in leaf shape and size observed among *B*. ×*ottawensis* hybrids. Among hybrid accessions, leaves vary in shape (ovate, oblong, or obovate), size (2–6 cm long and 1–4 cm wide), and margins (entire to >30 serrations).

**Fig. 2.**
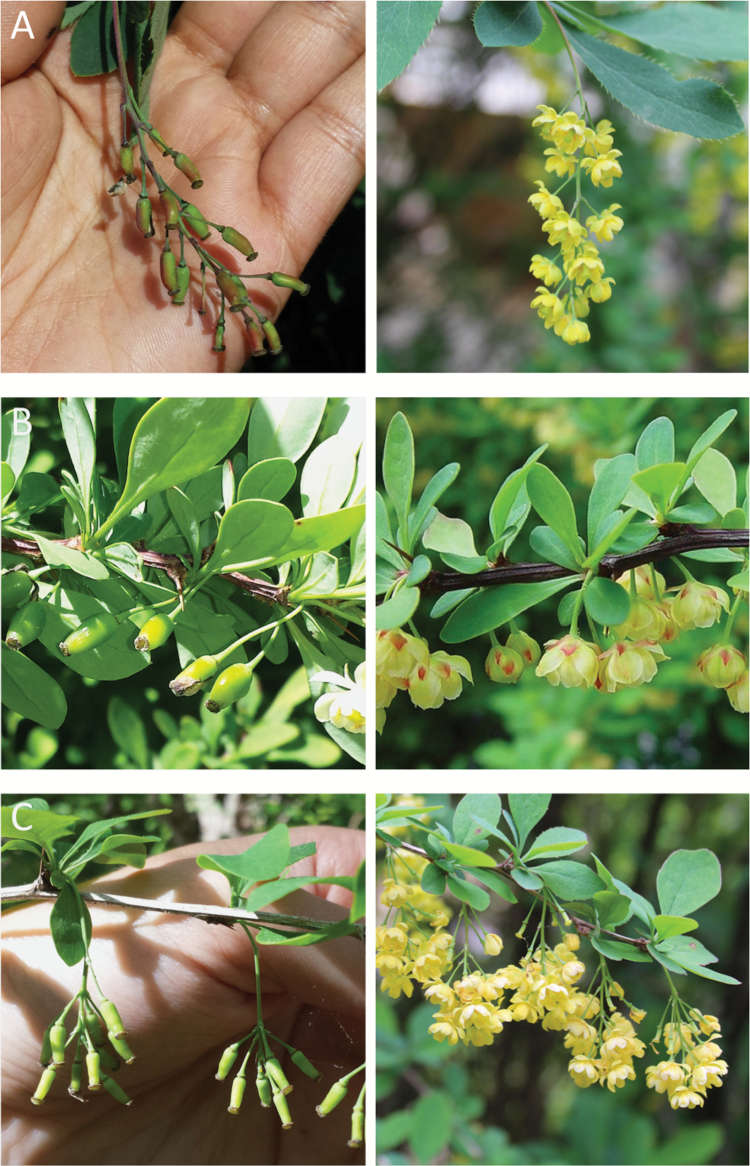
Infloresence types of the three *Berberis* taxa in the study. (A) 5–8 cm long pendant racemes with bright yellow flowers typical of *B*. *vulgaris*; (B) 1–2 cm long inflorescences with mostly solitary flowers typical of *B*. *thunbergii*; and (C) truncated pendant racemes with 5–12 bright yellow flowers typical of *B*. ×*ottawensis*.

Naturally occurring individuals of three barberry taxa, *B. vulgaris* L. (common or European barberry), *B. thunbergii* DC. (Japanese barberry), and *B.* ×*ottawensis* C.K. Scheid, were collected from Mass Audubon’s Lime Kiln Farm Wildlife Sanctuary in Sheffield, MA, for use in this study. *Berberis vulgaris* was first introduced to North America by European settlers during the 17th century (Grieve, 1931; Gleason and Cronquist, 1963) and is now considered an invasive species throughout many regions of the USA, including New England (Mehrhoff *et al*., 2003). These upright, perennial shrubs grow up to 3 m tall, display 2–5 cm long obovate to obovate–oblong leaves with highly serrated margins (>50 serrations), and have 5–8 cm long pendant racemes of bright yellow flowers (Gleason and Cronquist, 1963; Mehrhoff *et al*., 2003). *Berberis thunbergii*, first introduced to North America as an ornamental plant from Japan in 1875 (Steffey, 1985), is also now considered an invasive species throughout New England, the Midwest, and eastern states. It is a relatively smaller shrub, ranging from 0.5 m to 2.5 m tall, that displays 1.3–3.8 cm long entire leaves and 1–2 cm long inflorescences with few umbellate but mostly solitary flowers (Gleason and Cronquist, 1963; Mehrhoff *et al*., 2003; Haines, 2011). The third taxon, *B. ×ottawensis*, is the nothospecies resulting from the interspecific hybridization of *B. vulgaris* and *B. thunbergii* (Rehder, 1949). This hybrid is intermediate in height and leaf size between the two parental species, with either entire or moderately serrated leaf margins and truncated pendant racemes bearing 5–12 bright yellow flowers (Mehrhoff *et al*., 2003; Connolly *et al.*, 2013). Representative images of the leaf morphologies and inflorescence types of the three taxa are shown in Figs 1 and 2, respectively.

In June 2014, a field survey was conducted to document and characterize the natural populations of *B. vulgaris*, *B. thunbergii*, and *B.* ×*ottawensis* growing within an ~7 ha area section of the Lime Kiln Farm Wildlife Sanctuary. All *Berberis* spp. plants located within the study area were keyed to species based on the following morphological parameters: plant height, growth habit, leaf morphology, and inflorescence/flower morphology. Of the nearly 1000 plants keyed to species, 190 were selected for subsequent propagation, genotyping, and disease phenotyping, comprising 22 *B. vulgaris*, 27 *B. thunbergii*, and 141 putative *B.* ×*ottawensis* accessions. Prior to sampling, these 190 plants were assigned unique IDs, labeled with metal tags, and geo-referenced using a Garmin eTrex Vista HCx GPS unit.

In August 2014, 1-year-old semi-hardwood cuttings were taken from each of the 190 selected accessions for clonal propagation via rooting at the MacFarlane Research Greenhouses at the University of New Hampshire (UNH) in Durham, NH. To propagate, the proximal end of a 10–16 cm stem cutting was dipped in dry Hormodin-1 (0.1% indole-3-butyric acid) rooting hormone powder and inserted into moistened vermiculite. The set cuttings were maintained at 30–35 ºC, and light misting was provided every 5–6 min to maintain high relative humidity throughout the propagation period. Once rooted, cuttings were transplanted into black plastic pots (11.5 cm diameter × 6.5 cm tall) filled with PRO-MIX HP growth media and maintained in the greenhouse for tissue sampling and disease phenotyping.

### DNA isolation and genotyping by sequencing

Based on relative propagation success, a random subset of 80 collected accessions were selected for genotypic characterization, including 8 *B. vulgaris*, 9 *B. thunbergii*, and 63 putative *B.* ×*ottawensis* genotypes (see Supplementary Fig. S1 at *JXB* online). To increase the sampled genetic diversity and thus the confidence in calling true species-specific variants (i.e. variants polymorphic between *B. vulgaris* and *B. thunbergii* but monomorphic within each species), three additional *B. vulgaris* and four additional *B. thunbergii* accessions were collected from other sites (Randall Road, Lee, NH; Adams Point Road, Durham, NH; Piscataqua Road, Durham, NH; and Agronomy Road, Storrs, CT) and included for genotyping (Supplementary Table S1.). For all 87 accessions, genomic DNA (gDNA) was extracted from ~100 mg of lyophilized leaf tissue using a modified cetyltrimethylammonium bromide (CTAB) method (Doyle, 1987). Prior to genotyping by sequencing (GBS) library preparation, the isolated gDNA was purified using Zymo Research’s Genomic DNA Clean & Concentrator™ (Catalog # D4011), following the manufacturer’s protocol.

Reduced representation libraries were constructed using the two-enzyme (*Pst*I–*Msp*I) GBS protocol described by Poland *et al.* (2012) and sequenced via 150 bp paired-end (PE) reads on an Illumina HiSeq 2500 at the Hubbard Center for Genome Studies, UNH. Raw FASTQ files were generated using CASAVA 1.8.3 and analyzed using the *de novo* (i.e. reference-free) bioinformatics pipeline GBS-SNP-CROP (Melo *et al.*, 2016). In the first stage of the pipeline, all raw reads were parsed, quality trimmed, and demultiplexed into individual read pairs per genotype. A mock reference was constructed using the high-quality PE reads from all 63 *B.* ×*ottawensis* accessions, and putative variants, both single nucleotide polymorphisms (SNPs) and bi-allelic indels, were identified by aligning the high-quality PE reads of all 63 *B.* ×*ottawensis* accessions to the mock reference, following the pipeline’s recommended parameters. Using SAMTools (Li *et al.*, 2009), only those read pairs possessing high mapping quality (*q*>30), exhibiting proper pair orientation, and showing no supplementary alignments were retained for variant calling. The parameters used for variant calling followed the pipeline recommendations for diploid species, except that the -mnAvgDepth parameter was increased to 7 to enhance accuracy, at the expense of total marker number. Complete details of the GBS-SNP-CROP command lines used in this study, including all specified pipeline parameters, are provided in Supplementary Text S1.

The 11 *B. vulgaris* and 13 *B. thunbergii* accessions included in the GBS library were genotyped by mapping their high-quality PE reads to the *B.* ×*ottawensis* mock reference and calling alleles only for those marker positions identified as segregating among the natural population of *B*. ×*ottawensis* lines. For all downstream genetic diversity and pedigree analyses, only those variants located within centroids (i.e. consensus GBS fragments) containing single markers (hereafter referred to as simplex markers) were retained. All parsed, high-quality PE reads are deposited in the National Center for Biotechnology Information (NCBI) Short Read Archive (SRA). Individual barcode assignments, species information, detailed sequencing statistics, and assigned SRA numbers for the 87 genotyped accessions can be found in Supplementary Table S1.

### Characterization of genetic diversity

To characterize the genetic diversity both within and among the populations of the three *Berberis* taxa present in the Lime Kiln Farm Wildlife Sanctuary, variants (or markers) were first characterized based on average read depth (D), the percentage of homozygotes (Homo), the percentage of heterozygotes (Hetero), and the proportion of missing genotype calls (NA). The software GenAIEx 6.5.01 (Peakall and Smouse, 2006) was then used to generate descriptive population genetic parameters, such as the numbers of effective alleles (*N*_E_), the minor allele frequencies (MAF), the observed heterozygosities (*H*_O_), the unbiased expected heterozygosities (*H*_E_), the inbreeding coefficients (*F*_IS_), and the measure of interspecific genetic structure (*F*_ST_). Interspecific genetic structure was also assessed via principal component analysis (PCA) through the dudi.pca() function in R (package ‘adegenet’) (Jombart and Ahmed, 2011).

### Pedigree inference for *B. ×ottawensis* individuals

To infer the pedigrees (e.g. F_1_, BC_1_, etc.) of the 63 genotyped *B.* ×*ottawensis* hybrid individuals, species-specific markers (i.e. variants polymorphic between *B. vulgaris* and *B. thunbergii* but monomorphic within each species) were identified. To ensure the informativeness of such markers across the population, we excluded all SNPs and indels for which there were >25% missing data. Further filtering was applied to retain only 459 high confidence, species-specific markers that exist in alternate homozygous states between all genotyped accessions of the two parental species, including the additional seven accessions from sites other than Lime Kiln (Supplementary Table S1). For those 459 high confidence, species-specific markers, alleles specific to *B. thunbergii* are referred to as ‘Bt’ alleles and alleles specific to *B. vulgaris* are referred to as ‘Bv’ alleles.

Pedigree inferences for the 63 *B*. ×*ottawensis* accessions were made based on the percentage compositions of Bt homozygous loci, Bv homozygous loci, and Bv/Bt heterozygous loci within each individual. Because the pedigree analysis involves only species-specific markers, the theoretical expectation for true F_1_ hybrids is 100% heterozygosity across all loci (i.e. 100% Bv/Bt). Due to both sampling bias and inherent genotyping error, however, 100% heterozygosity is not expected in empirical data; thus in this study, *B*. ×*ottawensis* individuals were considered F_1_ hybrids if the observed percentage of Bv/Bt heterozygosity across all 459 high-confidence species-specific markers was >95% and the combined percentage of Bv homozygous and Bt homozygous loci was <5%. More complex pedigrees were similarly inferred, under the simplifying assumption of independent segregation. A reference table of expected proportions of Bt homozygous, Bv homozygous, and Bv/Bt heterozygous loci for possible hybrid pedigrees, up to four generations, is provided in Supplementary Table S2.

### Disease phenotyping

In April and May 2015, rooted cuttings of the 87 genotyped accessions were phenotyped for their responses to *Pg* at the USDA-ARS Cereal Disease Laboratory (CDL) in St. Paul, MN, using the standard protocol (Cotter, 1932) for testing barberry varieties for release to regulated states, as per CDL’s contract with the USDA Animal and Plant Health Inspection Service. If scored as resistant at the CDL, the disease reactions of the 63 genotyped *B.* ×*ottawensis* individuals were verified via an independent round of testing at UNH in April and May 2016. For all disease phenotyping, newly emerged leaves were inoculated with germinated *Pg* basidiospores by suspending overwintered, telia-laden straw of naturally infected *Elymus repens* (L.) Gould over barberry plants in an inoculation chamber cycling between 18 °C (light, 14 h) and 16 °C (dark, 10 h). The *Pg*-infected stems of *E. repens* were collected in 2013 in St. Charles, MN, where a population of *Pg* is alternating between *B. vulgaris* and *E. repens* (Y. Jin, unpublished) and are part of CDL’s source inoculum for barberry testing. In that region, *E. repens* is commonly infected by two *Puccinia* spp. (*P. graminis*, the causal organism of stem rust, and *P. coronata*, the causal organism of crown rust), but the two are easily distinguished on the basis of telia and teliospore morphologies (Cummins, 1971). Also, given the fact that the alternate host for *P. coronata* is *Rhamnus* spp. rather than *Berberis* spp., the natural inoculum used in this study was certainly *Pg*.

Two days post-inoculation, plants were moved to a growth chamber or greenhouse cycling between 20 ± 2 °C (light, 14 h) and 18 ± 2 °C (dark, 10 h) for further incubation. Infections were scored visually 14 d after inoculation, when mature aecia developed on the susceptible *B. vulgaris* check. Individual plants were scored as *Pg* susceptible if more than five pycnia were seen to develop on the upper surfaces of individual leaves and mature aecia were seen to develop on the lower surfaces. Individuals were scored as *Pg* resistant if the inoculation failed to produce visual symptoms, apart from minor flecking. Individuals were scored as intermediate if any of the following were observed: hypersensitive reactions, including chlorosis and/or necrosis; leaf reddening; or fewer than five pycnia with no associated aecia. If the susceptible checks failed to exhibit clear infection, all accessions in that group were subjected to another round of inoculation and rescored. Because of the inherent spatial heterogeneity of basidiospore ejection during inoculation, *Pg* susceptibility is called with higher confidence than *Pg* resistance. Therefore, all *B.* ×*ottawensis* individuals scored as resistant were screened a second time to reduce the chance of false negatives (Supplementary Table S3).

### Testing maternal inheritance of *Pg*-NHR

To test whether or not the *Pg*-NHR of *B. thunbergii* observed segregating in the population of Lime Kiln hybrids is maternally inherited, an independent population of 129 F_1_*B. ×ottawensis* full sibs was developed via a controlled cross between *B. vulgaris* accession ‘Wagon Hill’ (female parent) and *B. thunbergii* accession ‘UCONN1’ (pollen parent). The hybrid status of all progeny was validated via GBS, and their reactions to *Pg* were evaluated at UNH in April and May 2016, following the method described above.

## Results

### Relative abundance and phenotypic characterization of *Berberis* taxa at the study site

The field survey conducted to characterize the natural populations of *B. vulgaris*, *B. thunbergii*, and *B.* ×*ottawensis* growing within the Lime Kiln Farm Wildlife Sanctuary in Sheffield, MA, revealed wide distribution of all three barberry taxa within the study area (Supplementary Fig. S1). The combined population size of the three taxa was estimated to be ~1000 individuals, spread over an area of ~7 ha. *Berberis thunbergii* (~600 individuals) was observed to outnumber the other two taxa, although *B.* ×*ottawensis* and *B. vulgaris* plants were also observed in significant numbers (~200 each). While morphological variation was observed within the populations of all three taxa, the most pronounced variation within a taxon was observed among the *B.* ×*ottawensis* hybrids, especially in the diagnostic characteristics of plant height (0.5–3 m tall), leaf size (2–6 cm long and 1–4 cm wide), and leaf margin (entire to >30 serrations). While some *B.* ×*ottawensis* individuals were as tall as or even taller than mature *B. vulgaris* plants, their leaves were often smaller than those of *B. vulgaris* and/or their margins had far fewer serrations. In contrast, other hybrids largely resembled *B. thunbergii*, in the sense of being relatively short (0.5–1 m tall) and having non-serrated leaves, but bore 5–12 bright yellow flowers on truncated pendant racemes typical of the nothospecies.

### Variant calling

GBS generated a total of 381 million 150 bp PE reads for the 87 accessions selected at random from the study site. After quality parsing and demultiplexing, the average numbers of high-quality reads per genotype retained by the GBS-SNP-CROP pipeline were 2.8, 3.5, and 4.8 million PE reads for *B. thunbergii*, *B. vulgaris*, and *B.* ×*ottawensis*, respectively (Supplementary Table S1). Using high-quality PE reads from all 63 *B.* ×*ottawensis* individuals, we generated a mock reference comprised of 143213 centroids (i.e. consensus GBS fragments), with a total length of ~25 Mbp. In total, the pipeline identified 23091 putative variants, including 20799 SNPs (average depth D_SNPs_=26.71) and 2292 bi-allelic indels (D_indels_=25.22), and the percentage of missing data was low (4%). After filtering, the final set of 2369 simplex markers (i.e. SNPs or indels located within centroids containing a single polymorphic site) exhibited higher depth (D=66.67) but a similar pattern of heterozygosity, homozygosity, and missing data characteristic of the entire data set (Table 1).

**Table 1.** Summary data characterizing the variants called for 63 accessions of *B. ×ottawensis* collected from the Lime Kiln Farm Wildlife Sanctuary in Sheffield, MA

Type^*a*^	N^*b*^	D^*c*^	%Het^*d*^	%Hom^*e*^	%NA^*f*^
All markers					
SNP	20799	48.74	55.89	40.22	3.89
Indel	2292	46.45	54.79	40.69	4.52
Both	23091	48.51	55.78	40.26	3.96
Simplex markers only					
SNP	2164	67.55	50.03	46.15	3.81
Indel	205	57.42	55.02	39.20	5.77
Both	2369	66.67	50.46	45.55	3.98

^*a*^The type of variant called by the *de novo* GBS-SNP-CROP pipeline (v.2.1), either SNPs or indels.

^*b*^The number of variants, by type, called after imposing all recommended genotyping criteria for diploid species.

^*c*^The average read depth, by variant type.

^*d*^The percentage of heterozygous loci throughout the population.

^*e*^The percentage of homozygous loci throughout the population.

^*f*^The percentage of missing cells (i.e. no genotype assigned for a given variant–accession combination)

### Assessment of genetic diversity

Genetic diversity analyses were performed within and among the three populations of barberry taxa at Lime Kiln using the 2369 simplex markers described above (see Table 2). In terms of intraspecific genetic diversity, the percentage of polymorphic loci was nearly 100% within the *B.* ×*ottawensis* subpopulation, a result which reflects the hybrid nature of these individuals and supports the high level of unbiased expected heterozygosity (*H*_E_=0.375) estimated for the nothospecies. The percentages of polymorphic loci were relatively lower for both of the parental species (21.7% for *B. vulgaris* and 41.0% for *B. thunbergii*), and the lowest value of *H*_E_ was found for *B. vulgaris* (*H*_E_=0.088). Like this low value of *H*_E_, the highly negative value of *F*_IS_ (–0.24) for *B. vulgaris* is unsurprising in light of the severe genetic bottleneck presumed for this species during its colonial introduction from Europe to North America.

**Table 2.** Population parameters characterizing the genetic diversity among and within the three sampled subpopulations of barberry taxa, based on 2369 simplex markers

Taxon	*n* ^*a*^	%POL^*b*^	H_O_^*c*^	H_E_^*d*^	*F*_IS_^*e*^
*B. thunbergii*	9	40.95	0.152	0.153	–0.065
*B. vulgaris*	8	21.74	0.104	0.088	–0.243
*B.* ×*ottawensis*	63	99.96	0.462	0.375	–0.085
Means		54.22	0.239	0.205	–0.131

^*a*^The number of genotypes sampled, by species.

^*b*^The percentage of polymorphic loci within each species.

^*c*^The observed heterozygosity.

^*d*^The unbiased expected heterozygosity.

^*e*^The inbreeding coefficient.

In general, the low level of inbreeding observed for all three species (average *F*_IS_= –0.131) is an understandable consequence of their outcrossing physiologies (Lebuhn and Anderson, 1994). The high value of the interspecific genetic structure between *B. vulgaris* and *B. thunbergii* (*F*_ST_=0.738) indicates a robust population structure in spite of co-location, a structure that is probably maintained due to flowering time differences between the parental species, with *B. thunbergii* flowering 2–4 weeks earlier than *B. vulgaris* in the region (Connolly *et al.*, 2013). Both the overall taxa-based population structure and the relative genetic diversity within taxa are well captured by a PCA (Supplementary Fig. S2), in which the first two axes account for >89% of the genetic diversity.

### Inferred pedigrees of the *B. ×ottawensis* individuals

From a total of 2369 simplex markers, 459 high-confidence, species-specific variants (i.e. SNPs or indels polymorphic between the two parental species but monomorphic within) were retained and used for pedigree analysis. Using this reduced set of parental species-informative markers, we inferred the generic pedigrees of the 63 genotyped *B*. ×*ottawensis* individuals based on the percentage compositions of Bt homozygous, Bv homozygous, and Bt/Bv heterozygous loci within each individual, where ‘Bt’ designates an allele specific to *B. thunbergii* and ‘Bv’ designates an allele specific to *B. vulgaris*. Of the 63 genotyped *B.* ×*ottawensis* individuals, 53 (84%) were found to be heterozygous (Bt/Bv) at ≥95% of the 459 loci; hence the vast majority of *B. ×ottawensis* individuals appear to be true, first-generation (F_1_) hybrids at the study site (Table 3). Comparatively smaller numbers of *B. ×ottawensis* individuals appear to be later generation hybrids (e.g. backcrosses to parental species, etc.; see Table 3). In addition to the expected proportions of Bt homozygous, Bv homozygous, and Bt/Bv heterozygous loci for various possible pedigrees (Supplementary Table S2), the full data set with actual proportions and inferred pedigrees is presented in Supplementary Table S3.

**Table 3.** Inferred pedigrees and observed reaction types of the 63 genotyped *B.* ×*ottawensis* accessions, based on 459 species-specific markers

Inferred pedigree^*a*^	S^*b*^	I^*c*^	R^*d*^	F^*e*^
Bt/Bv=F_1_	26	8	17	2
F_1_/Bv	1	0	0	0
F_1_/Bt//Bv	1	1	0	0
F_1_/Bv//Bv	1	0	0	0
F_1_/Bv//Bt	1	0	0	0
F_1_/Bt//Bv///Bt	0	2	0	0
F_1_/Bv//Bt///Bv	1	0	0	0
F_1_/Bt//Bt///Bt////Bv	2	0	0	0
Total	33	11	17	2

^*a*^Accession pedigrees are inferred based on observed proportions of homozygous and heterozygous loci, considering only species-specific markers and assuming independent segregation (Supplementary Table S2). Bt designates a *B. thunbergii* parent, Bv designates a *B. vulgaris* parent, and F_1_ designates the *B.* ×*ottawensis* F_1_ hybrid. Within each pedigree, one slash (‘/’) indicates the first cross, two slashes (‘//’) indicate the second cross, and so forth. For example, pedigree ‘A/B//C///D’ indicates that A was first crossed with B, their offspring was crossed with C, and that offspring was crossed with D.

^*b*^The number of *Pg*-susceptible genotypes.

^*c*^The number of intermediate genotypes.

^*d*^The number of *Pg*-resistant genotypes.

^*e*^The number of failed inoculations (i.e. no disease phenotype).

### Reaction to *Puccinia graminis* inoculation

**Fig. 3.**
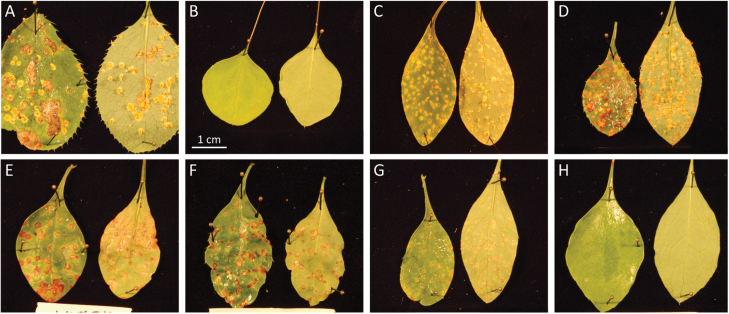
Representative responses of *B. vulgaris*, *B. thunbergii*, and *B.* ×*ottawensis* accessions to *P. graminis* inoculation, using overwintered teliospores of naturally infected *E. repens* under controlled conditions. All photos were taken 14 d post-inoculation. (A) Susceptible reaction of *B. vulgaris* accession ‘LK-070’, showing pycnia on the upper surfaces of leaves and well-developed aecia on the lower surfaces of leaves. (B) Resistant reaction of *B. thunbergii* accession ‘LK-107’, showing no visual symptoms. (C, D) Susceptible reactions of *B.* ×*ottawensis* accessions ‘LK-165’ and ‘LK-160’, showing well-developed pycnia and aecia. (E–G) Intermediate reactions on *B.* ×*ottawensis* accessions ‘LK-074’, ‘LK-121’, and ‘LK-137’, showing sites of red or brown necrosis. (H) Resistant reaction of *B.* ×*ottawensis* accession ‘LK-015’, with no visual symptoms. White scale bar=1 cm.

To determine disease responses to *Pg*, individual propagated accessions were inoculated using overwintered telia of *Pg* found on naturally infected *E. repens*. Of the 190 individuals collected from the field, inoculation was successful for 122 accessions, with the other 68 accessions dying during either shipment, propagation, or handling in the greenhouse. One week after inoculation, pycniospores began to develop on the upper surfaces of young leaves of both *B. vulgaris* and susceptible *B.* ×*ottawensis* individuals, whereas resistant *B.* ×*ottawensis* individuals showed varying responses, ranging from no visual symptoms (similar to *B. thunbergii*) to the development of sparse brown flecking. Two weeks after inoculation, accessions of *B. vulgaris* exhibited a clear susceptible reaction, with well-developed mature aecia visible on the lower leaf surfaces (Fig. 3A). In contrast, accessions of *B. thunbergii* developed either no symptoms at all or small, sparse flecking (Fig. 3B), and accessions of *B.* ×*ottawensis* showed varying responses, ranging from *B. vulgaris*-like full susceptibility (Fig. 3C) to *B. thunbergii*-like resistance (Fig. 3H). Various intermediate disease responses of some *B.* ×*ottawensis* accessions included aecial development in the presence of red necrotic islands (Fig. 3D), reddish or brown necrotic lesions with no aecial development (Fig. 3E, F), and flecking (Fig. 3G). As summarized in Table 4, all *B. vulgaris* accessions exhibited clear susceptible reactions and all *B. thunbergii* accessions exhibited clear resistant reactions. Of the 105 *B.* ×*ottawensis* accessions successfully tested for disease response to *Pg*, 54 (52%) exhibited clear susceptible reactions with well-developed pycnia and mature aecia, and 37 (35%) accessions exhibited clear resistant reactions with either no visual symptoms or sparse, small flecking. The remaining 14 accessions (13%) exhibited various intermediate responses, usually involving <5 pycnia, no aecial development, and associated hypersensitive-like reactions, including chlorosis, necrosis, and leaf reddening.

**Table 4.** Summary of reactions to *P. graminis* by the 122 Lime Kiln accessions of three barberry taxa as well as the derived population of 129 *B.* ×*ottawensis* full sibs

	S^*a*^	I^*b*^	R^*c*^
Taxa collected from Lime Kiln			
*B. thunbergii*	0 (0%)	0 (0%)	13 (100%)
*B. vulgaris*	4 (100%)	0 (0%)	0 (0%)
*B.* ×*ottawensis*	54 (52%)	14 (13%)	37 (35%)
‘Wagon Hill’/‘UCONN1’ full sibs			
*B.* ×*ottawensis*	81 (63%)	23 (18%)	25 (19%)

^*a*^The number and percentage of *Pg*-susceptible genotypes.

^*b*^The number and percentage of intermediate genotypes.

^*c*^The number and percentage of *Pg*-resistant genotypes.

The population of F_1_*B. ×ottawensis* full sibs derived from the cross between *B. vulgaris* accession ‘Wagon Hill’ and *B. thunbergii* accession ‘UCONN1’ was similarly tested for response to *Pg.* As within the natural population of *B. ×ottawensis* hybrids, varying responses ranging from apparent immunity to severe susceptibility were observed to segregate among the full sibs. Of the 129 full sib lines tested for disease response to *Pg*, 81 (63%) exhibited a clear susceptible reaction, 23 (18%) exhibited an intermediate response, and 25 (19%) exhibited a resistant reaction (Table 4).

## Discussion

As the most common form of genetic resistance, NHR has the potential to provide broad-spectrum, durable resistance to many plant pathogens, including the causal organism of wheat stem rust. Unfortunately, the genetic mechanisms underlying *Pg*-NHR are poorly understood, in part due to the inherent challenge of developing a genetically tractable system in which genes controlling *Pg*-NHR segregate. In this study, we investigated the disease response of an interspecific hybrid between *Pg*-resistant *B. thunbergii* and *Pg*-susceptible *B. vulgaris*, and demonstrated the viability of this unique pathosystem to begin characterizing and mapping the gene(s) underlying the putative *Pg*-NHR of *B. thunbergii*.

### The apparent non-host resistance of *B. thunbergii* to *P. graminis* segregates in the interspecific hybrid *B. ×ottawensis*

The natural population of *B. ×ottawensis* hybrids screened for disease response in this study was found to segregate for resistance to *Pg*. Specifically, 52% of the successfully screened hybrid accessions exhibited *B. vulgaris*-like susceptibility, 35% showed clear *B. thunbergii*-like resistance, and the remaining 13% showed varying intermediate reactions. GBS analysis performed on a random subset of 63 of these phenotyped *B. ×ottawensis* accessions showed that 53 were true, first-generation (F1) hybrids, among which a similar proportion of susceptible (51%), resistant (33%), and intermediate (16%) reactions were observed (Table 3). These results demonstrate that the *Pg*-NHR observed in *B. thunbergii* segregates in a population of first-generation (F_1_) interspecific hybrids with *Pg*-susceptible *B. vulgaris*. Therefore, the gene(s) underlying *Pg*-NHR in *B. thunbergii* are, in theory, mappable in an F_1_ population derived from the controlled hybridization of *Pg*-susceptible *B. vulgaris* and *Pg*-resistant *B. thunbergii*.

Over the past decade, in light of growing global concern about the wheat rusts, a number of efforts have been mounted to understand NHR to rust pathogens using various model and non-model plants. Many plant species, including *A. thaliana*, *Brachypodium distachyon*, rice, barley, and cowpea (Ayliffe *et al.*, 2011; Cheng *et al.*, 2012, 2013; An *et al.*, 2016; Dawson *et al.*, 2016; Li *et al.*, 2016; Zhao *et al.*, 2016), have been used to study NHR to *P. striiformis* f. sp. *tritici* (*Pst*), the causal organism of wheat stripe rust. In contrast, *Pg*-NHR has thus far been studied only in rice (Ayliffe *et al.*, 2011), as distinct from the studies of intermediate *Pg* resistance conducted in barley and *B. distachyon* (Figueroa *et al.*, 2013; Dracatos *et al.*, 2014). Given the evolutionary relationship between the *Berberis* genus and *Pg* prior to its host expansion to the grasses, the findings of this study suggest that the interspecific hybrid *B. ×ottawensis* may have value as an alternative, novel system for mapping and gaining insight into the genetic mechanism(s) underlying resistance to this complex pathogen.

### *Pg*-NHR in *B. thunbergii* probably involves more than one nuclear gene

From the practical standpoint of breeding for improved resistance to biotic factors, the central questions regarding NHR concern the nature and modes of inheritance of the underlying genes. In the case of maize NHR to the rice bacterial streak pathogen *Xanthomonas oryzae* pv. *oryzicola*, Zhao *et al.* (2004) reported that the resistance is mediated by a single gene, designated *Rxo1*. If a single gene governs *Pg*-NHR in *B. thunbergii*, the segregation of resistance among F_1_ hybrids documented in this study suggests that the underlying resistance gene may exhibit dominance and exist in a heterozygous state within *B. thunbergii*. In such a case, however, independent assortment during meioses would invariably result in homozygous *Pg*-susceptible *B. thunbergii* progeny. To date, no accession of *B. thunbergii* tested by the CDL has shown susceptibility to *Pg*; thus a single gene governing *Pg*-NHR in *B. thunbergii* is unlikely. The range of disease responses observed in this study, including a complete lack of visual symptoms (i.e. immunity), various intermediate-level reactions, and full susceptibility (Fig. 3), also suggests that the *Pg*-NHR of *B. thunbergii* is probably governed by more than a single gene.

Segregation of resistance within a natural population of F_1_ hybrids could also be explained if the inheritance of NHR is non-nuclear. If the *Pg*-NHR of *B. thunbergii* is transmitted via the cytoplasm, all offspring should exhibit a disease response similar to that of the maternal plant. Under natural conditions, such as those at the Lime Kiln Farm Wildlife Sanctuary, the relatively restricted gene flow between *B. thunbergii* and *B. vulgaris* is assumed to be bi-directional, meaning both species have an equal chance to serve as the female parent of *B. ×ottawensis* hybrids. If *Pg*-NHR is cytoplasmically inherited, all hybrid progeny obtained from a *B. thunbergii* mother plant are expected to be resistant, and all hybrids obtained from a *B. vulgaris* mother plant are expected to be susceptible. In this study, we found that ~50% of the Lime Kiln F_1_ hybrids exhibit a susceptible reaction, suggesting that the *Pg*-NHR of *B. thunbergii* may indeed be cytoplasmically inherited. To test this hypothesis, a population of 129 F_1_*B. ×ottawensis* full sibs was developed via a controlled cross between *B. vulgaris* (female parent) and *B. thunbergii* (pollen parent) and screened for disease response to *Pg*. Under this scenario, all F_1_ hybrids would be expected to exhibit *B. vulgaris*-like susceptibility to *Pg*; yet clear segregation in disease response was observed (Table 4), indicating that the *Pg*-NHR of *B. thunbergii* is not transmitted via the cytoplasm.

### *Pg*-resistant barberry species may be epidemiologically relevant to *Pg* evolution by virtue of their hybrid progeny

It is well established that naturalized populations of *B. thunbergii* and *B. vulgaris* are widespread throughout New England (Connolly *et al.*, 2013; Hale *et al.*, 2015), to the extent that both are considered invasive species. This study shows that *B.* ×*ottawensis* is present throughout the Lime Kiln Farm Wildlife Sanctuary, and other recent studies report that this interspecific hybrid, assumed previously to be quite rare, is commonly found where the two parental species co-occur (Connolly *et al.*, 2013; Hale *et al.*, 2015). Once they are confirmed to be resistant to *Pg* by the USDA, *B. thunbergii* cultivars are propagated and sold as ornamental shrubs throughout the USA, as part of a multi-million dollar nationwide industry (Lubell *et al.*, 2008). While individual *B. thunbergii* genotypes may be deemed to pose no risk in terms of stem rust epidemiology, the results of this study raise a concern about the epidemiological risk of their progeny. Given the documented ability of *B. thunbergii* to naturalize and disperse, the prolific fruit set of many ornamental cultivars, the Herculean effort to purge the landscape of *Pg*-susceptible barberry plants in the 20th century, and the ongoing need to prevent sexual recombination of the stem rust pathogen, this study indicates a need to investigate and reconsider the epidemiological risk posed by *B. thunbergii*, by way of its interspecific hybrid with *B. vulgaris*. Specifically, in light of concerns around both invasiveness and *Pg* epidemiology, perhaps the minimum standard for new ornamental cultivars of *B. thunbergii* should be sterility, as pioneered by the recently patented cultivars 'UCONNBTCP4N', 'UCONNBTB039', 'UCONNBTB048', 'UCONNBTB113', and 'NCBT1' Sunjoy Mini Maroon™.

Beyond the USA, the highly diverse *Berberis* genus is distributed nearly worldwide, with centers of diversity in southern Asia as well as Central and South America (Ahrendt, 1961; Rounsaville and Ranney, 2010), and evidence of the alternate host’s role in current rust epidemics is mounting. In China, for example, ~250 *Berberis* spp. are found, accounting for nearly 50% of the species recorded globally (Ying and Chen 2001); and the sexual recombination of *Pst* observed on barberries has been implicated in the high genetic diversity of *Pst* in that country (Lu *et al.*, 2009; Mboup *et al.*, 2009; Sharma-Poudyal *et al.*, 2013; Zheng *et al.*, 2013; Wang *et al.*, 2016). Similarly, *Pg*-compatible *B. holstii* growing near wheat production areas in the highlands of eastern Africa may have played a role in the emergence of new virulence combinations in that region, including the rapidly diversifying Ug99 family of races (Singh *et al.*, 2015; Zhang *et al.*, 2017). In Iran, a country where the fruit of *B. vulgaris* is produced commerically on ~11000 ha (Rahimi-Madiseh *et al.*, 2017), *Pg* races of highly diverse virulence profiles were recovered from aecial samples collected from infected *B. vulgaris* plants, indicating that *Pg* can complete its sexual cycle in the region (Hansen *et al.*, 2013). Indeed, barberries grow widely throughout the mountainous areas of Central West Asia and North Africa, including countries in the Ug99 pathway, yet their exact epidemiological relevance remains unclear. While the present study focuses only on the two barberry species found in New England, one imported from Europe and the other from Japan, their natural hybrid and its complex response to *Pg* raises questions about the epidemiological role of presumably rust-resistant *Berberis* spp. worldwide. In short, the existence of interspecific hybrids may bring additional complications to the important work of understanding the contribution of *Berberis* spp. to global rust cycles.

### Future work

To build on the results of this study, we are developing an F_1_ bi-parental mapping population via a controlled cross between *B. vulgaris* accession ‘Wagon Hill’ and *B. thunbergii* accession ‘UCONN1’ in order to develop linkage maps for both parental species and begin mapping the gene(s) underlying *Pg*-NHR in *B. thunbergii*. To facilitate downstream dissection of potential quantitative trait loci, a reference genome for *B. thunbergii* is also in development. While it is possible, even likely, that the mechanisms governing *Pg*-NHR at the basidiospore stage (i.e. *Pg*’s infection of its sexual host) may not be relevant to those governing *Pg*-NHR at the aeciospore or urediospore stages (i.e. the spores which infect *Pg*’s asexual hosts, including wheat and other small grains), the ongoing, centuries-long fight against this complex and historic pathogen demands that all strategies be pursued. Ultimately, the hope is that characterization of *Pg*-NHR in *B. thunbergii*, a species closely related to the pathogen’s ancestral host, may provide information about the evolution of modern day heteroecious *Pg* and contribute insight into possible mechanisms of durable resistance in wheat.

## Supplementary data

Supplementary data are available at *JXB* online.

**Fig. S1.** Map of the Lime Kiln Farm Wildlife Sanctuary in Sheffield, MA.

**Fig. S2.** Principal components analysis showing the genetic structure of the three barberry subpopulations at the Lime Kiln Farm Wildlife Sanctuary.

**Table S1.** The 87 *Berberis* accessions used in the study, with passport information, genotypic data, and phenotypic features.

**Table S2.** Expected proportions of homozygous and heterozygous loci of hybrid accessions of various potential pedigrees.

**Table S3.** Disease reactions and observed proportions of homozygous and heterozygous loci within each of the 63 genotyped hybrid accessions.

**Text S1.** Detailed record of the GBS-SNP-CROP command lines used in this study, including all specified pipeline parameters.

Supplementary Table S1

Supplementary Table S2

Supplementary Table S3

Supplementary Figures S1-S2

supplementary Text
