# Supplementary material for: An interspecific barberry hybrid enables genetic dissection of non-host resistance to the stem rust pathogen Puccinia graminis
Source: J Exp Bot. 2018 Feb 26;69(10):2483–93. doi: 10.1093/jxb/ery066 (PMC5920301; doi:10.1093/jxb/ery066)
Supplement: Supplementary Figures S1-S2 [file ery066_suppl_supplementary_figures_s1-s2.pdf]

An interspecific barberry hybrid enables genetic dissection of non-host resistance to the stem rust pathogen *Puccinia graminis*

Radhika Bartaula, Arthur TO Melo, Bryan Connolly, Yue Jin, and Iago Hale

Supplementary Figures

Supplementary Figure S1. Map of the Lime Kiln Farm Wildlife Sanctuary in Sheffield, MA.

Supplementary Figure S2. Principal components analysis showing the genetic structure of the three barberry sub-populations at the Lime Kiln Farm Wildlife Sanctuary.

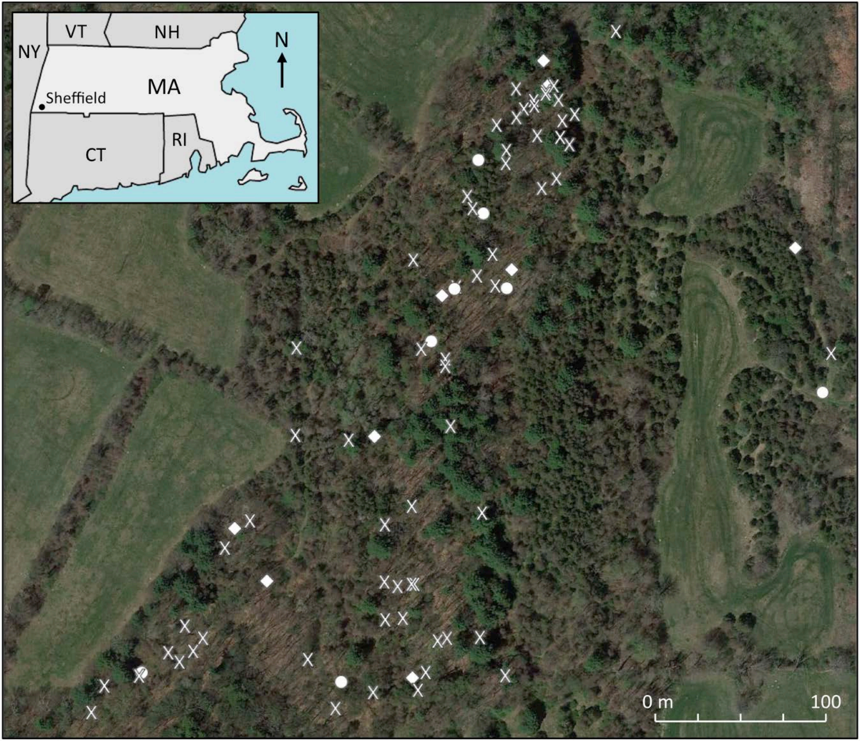

VT
NH
NY
N
MA
Sheffield
CT
RI
0 m
100

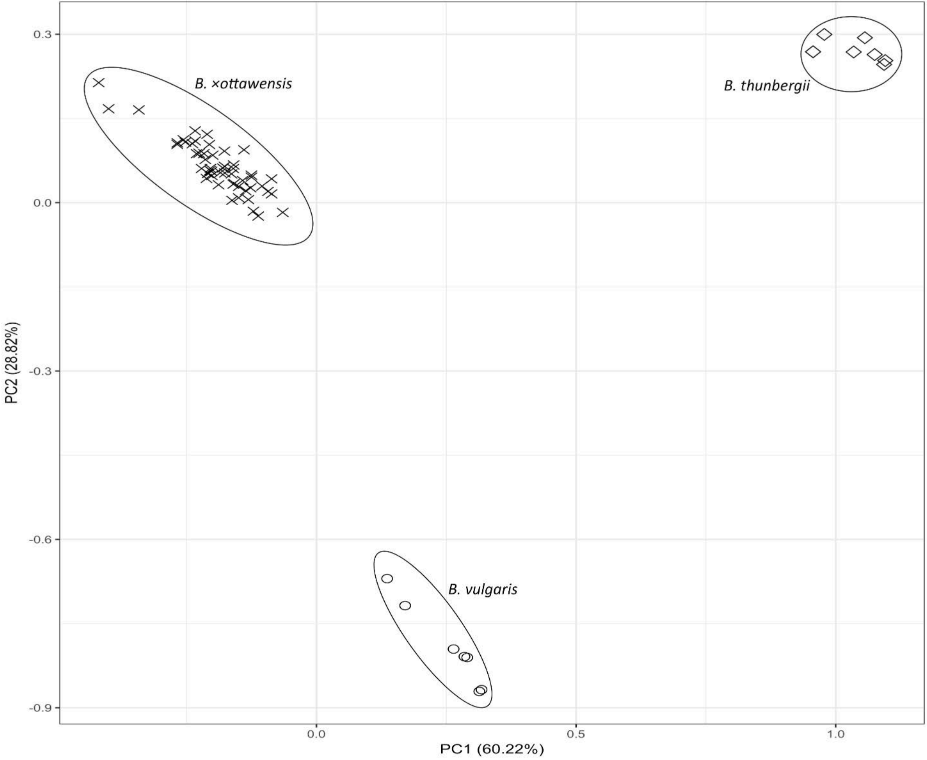
B. ×ottawensis
B. thunbergii
B. vulgaris
0.3
0.0
-0.3
-0.6
-0.9
0.0
0.5
1.0
PC1 (60.22%)
PC2 (28.82%)
