# Supplementary material for: An interspecific barberry hybrid enables genetic dissection of non-host resistance to the stem rust pathogen Puccinia graminis
Source: J Exp Bot. 2018 Feb 26;69(10):2483–93. doi: 10.1093/jxb/ery066 (PMC5920301; doi:10.1093/jxb/ery066)
Supplement: supplementary Text [file ery066_suppl_supplementary_text.pdf]

An interspecific barberry hybrid enables genetic dissection of non-host resistance to the stem rust pathogen *Puccinia graminis*

R Bartaula, A Melo, B Connolly, Y Jin, and I Hale

SUPPLEMENTARY MATERIALS

Text S1. Detailed record of the GBS-SNP-CROP command lines used in this study, including all specified pipeline parameters.

```
# GBS-SNP-CROP-1.pl
perl /path-to-workdir/GBS-SNP-CROP-1.pl -d PE -b barcodesIDs.txt -fq L001 -s 1 -e 48 -enz1 TGCA -enz2 CGG

# GBS-SNP-CROP-2.pl
perl /path-to-workdir/GBS-SNP-CROP-2.pl -d PE -fq L001 -t 10 -ph 33 -ad TruSeq3-PE.fa:2:30:10 -l 30 -sl 4:30 -tr 30 -m 32

# GBS-SNP-CROP-3.pl
perl /path-to-workdir/GBS-SNP-CROP-3.pl -d PE -b barcodesIDs.txt -fq L001

# GBS-SNP-CROP-4.pl
perl /path-to-workdir/GBS-SNP-CROP-4.pl -d PE -b barcodeID.txt -rl 150 -pl 32 -p 0.01 -id 0.93 -t 10 -MR MockRefName

# GBS-SNP-CROP-5.pl
perl /path-to-workdir/GBS-SNP-CROP-5.pl -d PE -b barcodeID.txt -ref MockRefName.MockRef.Genome.fasta -Q 30 -q 0 -f 2 -F 2308 -t 10 -Opt 0

# GBS-SNP-CROP-6.pl
perl /path-to-workdir/GBS-SNP-CROP-6.pl -b barcodeID.txt -out SNPs.summary.txt

# GBS-SNP-CROP-7.pl
perl /path-to-workdir/GBS-SNP-CROP-7.pl -in SNPs.summary.txt -out SNPs.call.txt -mnHoDepth0 5 -mnHoDepth1 20 -mnHetDepth 3 -altStrength 0.962 -mnAlleleRatio 0.25 -mnCall 0.75 -mnAvgDepth 7 -mxAvgDepth 200

# GBS-SNP-CROP-8.pl
perl /path-to-workdir/GBS-SNP-CROP-8.pl -in SNPs.call.txt -out SNP.Rmatrix -b barcodesIDs.txt -formats R
```
